# Development and Internal Validation of a Risk Score to Detect Asymptomatic Carotid Stenosis

**DOI:** 10.1016/j.ejvs.2020.11.029

**Published:** 2021-03

**Authors:** Michiel H.F. Poorthuis, Paul Sherliker, Dylan R. Morris, M. Sofia Massa, Robert Clarke, Natalie Staplin, Sarah Lewington, Gert J. de Borst, Richard Bulbulia, Alison Halliday

**Affiliations:** a Clinical Trial Service Unit and Epidemiological Studies Unit, Nuffield Department of Population Health, University of Oxford, Oxford, UK; b MRC Population Health Research Unit, Nuffield Department of Population Health, University of Oxford, Oxford, UK; c Department of Vascular Surgery, University Medical Centre Utrecht, Utrecht, The Netherlands; d Nuffield Department of Surgical Sciences, John Radcliffe Hospital, University of Oxford, Oxford, UK

**Keywords:** Atherosclerosis, Carotid artery stenosis, Ischaemic stroke, Prevention, Risk prediction model, Targeted screening

## Abstract

**Objective:**

Asymptomatic carotid stenosis (ACS) is associated with an increased risk of ischaemic stroke and myocardial infarction. Risk scores have been developed to detect individuals at high risk of ACS, thereby enabling targeted screening, but previous external validation showed scope for refinement of prediction by adding additional predictors. The aim of this study was to develop a novel risk score in a large contemporary screened population.

**Methods:**

A prediction model was developed for moderate (≥50%) and severe (≥70%) ACS using data from 596 469 individuals who attended screening clinics. Variables that predicted the presence of ≥50% and ≥70% ACS independently were determined using multivariable logistic regression. Internal validation was performed using bootstrapping techniques. Discrimination was assessed using area under the receiver operating characteristic curves (AUROCs) and agreement between predicted and observed cases using calibration plots.

**Results:**

Predictors of ≥50% and ≥70% ACS were age, sex, current smoking, diabetes mellitus, prior stroke/transient ischaemic attack, coronary artery disease, peripheral arterial disease, blood pressure, and blood lipids. Models discriminated between participants with and without ACS reliably, with an AUROC of 0.78 (95% confidence interval [CI] 0.77–0.78) for ≥ 50% ACS and 0.82 (95% CI 0.81–0.82) for ≥ 70% ACS. The number needed to screen in the highest decile of predicted risk to detect one case with ≥50% ACS was 13 and that of ≥70% ACS was 58. Targeted screening of the highest decile identified 41% of cases with ≥50% ACS and 51% with ≥70% ACS.

**Conclusion:**

The novel risk model predicted the prevalence of ACS reliably and performed better than previous models. Targeted screening among the highest decile of predicted risk identified around 40% of all cases with ≥50% ACS. Initiation or intensification of cardiovascular risk management in detected cases might help to reduce both carotid related ischaemic strokes and myocardial infarctions.

**What this paper adds**

Population level screening for asymptomatic carotid stenosis with duplex ultrasound is not recommended due to the low overall prevalence. Risk prediction models can be used to select particular individuals for targeted screening to detect asymptomatic carotid stenosis, allowing improved cardiovascular risk management to prevent complications.

## Introduction

Around 15%–20% of ischaemic strokes are caused by extracranial carotid stenosis,^1^ and such stenoses are also associated with an increased risk of coronary events and vascular death.^2^^,^^3^ Appropriate use of triple medical therapy (i.e., lipid lowering medication, antiplatelet drugs, and blood pressure lowering agents) in patients with clinically significant asymptomatic carotid stenosis (ACS), that is, ≥ 50% luminal narrowing, can help prevent strokes and myocardial infarctions (MIs). A study reporting on 387 patients with carotid stenosis related transient ischaemic attacks (TIAs) showed that less than half took statins and <60% were on antiplatelet therapy.^4^ This represents potential missed opportunities for stroke prevention.

The prevalence of moderate (≥50%) and severe (≥70%) ACS in the general population is low, with estimates of 2.0% and 0.5%, respectively; hence, population level screening for ACS with duplex ultrasound is not recommended in current guidelines.^5^, ^6^, ^7^, ^8^, ^9^

Risk scores to enable targeted screening of cases in populations with an elevated risk of ACS have been developed.^10^, ^11^, ^12^, ^13^, ^14^ A previous external validation of these established risk scores showed that the prediction model with the best predictive performance identified a group of cases at high risk of ACS with a number of participants needed to screen (NNS) of 21 to detect one case with ≥50% ACS when only people in the highest decile of predicted risk were screened.^15^ However, data used to develop these risk scores were based on participants who were recruited over two decades ago and important predictors of ACS, such as peripheral arterial disease (PAD), were not included in that model. A new risk score (the Prevalence of Asymptomatic Carotid Artery Stenosis [PACAS] risk score) was developed in a large contemporary screened population to predict ACS and to further reduce the NNS by targeted screening of those at highest risk of ACS.

## Materials and Methods

### Study population

Individual participant data from volunteers who attended commercial vascular disease screening clinics (run by Life Line Screening) between 2008 and 2013 in the USA and the UK were used. All individuals completed a standardised questionnaire, including questions about age, sex, height and weight, history of vascular disease (TIA, stroke, coronary heart disease [CHD]), hypertension, diabetes mellitus (DM), smoking status, and use of antiplatelet, antihypertensive, and lipid lowering medication. Standard blood pressure cuffs and sphygmomanometers were used, with systolic blood pressure (SBP) measured using a Doppler probe, and PAD was assessed by ankle brachial pressure index assessment.

Carotid duplex assessment was conducted by trained staff using dedicated vascular ultrasound instruments (GE LOGIQ e). Participants underwent bilateral examination of the carotid arteries with measurement of the highest peak systolic velocity (PSV) and end diastolic velocities of each common carotid and internal carotid artery.

A blood sample was provided by a subgroup of participants to measure plasma biochemistry using point of care testing methods (Alere Cholestech LDX system; Alere, Waltham MA, USA). Total cholesterol (TC) and high density lipoprotein (HDL) plasma levels were measured by enzymatic methods.

### Predicted outcomes

Two predicted outcomes were used: (1) moderate or severe ACS, i.e., estimated stenosis of 50%–100% (≥50%), based on a PSV of ≥125 cm/s on either side or 0 cm/s for occluded arteries; and (2) severe ACS, i.e., estimated stenosis of 70%–100% (≥70%), based on a PSV of ≥230 cm/s on either side or 0 cm/s for occluded arteries.

Mean degree of stenosis was determined according to the NASCET (North American Symptomatic Carotid Endareterectomy Trial) classification. If both sides showed ACS, patients were classified according to the greatest percentage of stenosis.

### Statistical analysis

#### Model development

Participants who provided a blood sample and who underwent duplex ultrasound of the carotid arteries were included in the present analyses. Age was categorised in four groups (<50 years, 50–59 years, 60–69 years, and ≥70 years), SBP in eight groups (<125 mmHg, 125–139 mmHg, 140–159 mmHg, and ≥160 mmHg in participants not using antihypertensives and in participants using antihypertensives), diastolic blood pressure (DBP) in three groups (≥85 mmHg, 75–84 mmHg, and <75 mmHg). Smoking status was dichotomised as current smoking *vs.* former or never smoking, and TC/HDL cholesterol ratio as ≥ 5 *vs.* < 5. For most predictors, the percentage of individuals with missing data was acceptable (<12%), except for measured DBP (31.8%) and waist circumference (WC) (34.9%) (Table S1; see Supplementary Material). Missing data were imputed via multiple imputation.

Multivariable logistic regression was performed to determine the relationships between predictors and the presence of ≥50% and ≥70% ACS. The same predictors as de Weerd *et al.*^14^ were considered (age groups, sex, current smoking, DM, history of stroke or myocardial infarction, SBP groups, DBP groups, and TC/HDL ratio group) and whether the addition of PAD, BMI, and WC improved the prediction was checked.^14^ Whether prediction was improved by adding a risk group for SBP and by taking antihypertensive use into account for SBP was also tested for.

Statistical performance of the prediction models was assessed by inspecting the discrimination and calibration. Discrimination assesses how well the prediction model differentiates between participants with and without ACS and was measured with the area under the receiver operating characteristic curve (AUROC). Calibration is an assessment of how closely the predictions of ACS of the model match the observed ACS risk in the data and was assessed graphically using calibration plots.

#### Internal validation and score chart

Internal validation assesses optimism and quantified the statistical performance of the prediction model. Bootstrap was used for internal validation.

Regression coefficients for the predictors were converted into points on a score chart. The risk of ≥50% and ≥70% ACS was calculated for the total points (sum scores). To convert regression coefficients to a score chart, the regression coefficient was multiplied by three (the smallest number while maintaining accurate prediction) and then rounded to the closed integer. If the scores for ≥50% and ≥70% were conflicting, the score for ≥50% was used.

#### Clinical application and reclassification measures

Test characteristics (prevalence, NNS, sensitivity, specificity, and positive and negative predictive values) of a targeted screening programme that uses the risk score for the selection of participants were calculated. Targeted screening programmes of the 10% and 20% highest risk participants were assessed. These thresholds were used to enable comparison with previously validated models.^15^

Reclassification measures were calculated to assess the ability of the PACAS risk score to correctly identify cases with and without ACS *vs.* the risk score of de Weerd *et al.*^14^ Integrated discrimination improvement (IDI), relative IDI, and category based net reclassification improvement were calculated.^16^^,^^17^ A *p* value < .05 was considered significant.

The study adhered to the Transparent Reporting of a multivariable prediction model for Individual Prognosis Or Diagnosis (TRIPOD) statement (Table S2; see Supplementary Material).^18^ Details about the statistical analysis can be found in Appendix S1. STATA version 15.1 (StataCorp, College Station, TX, USA) was used for all statistical analyses, and R version 3.5.1 was used to construct the figures.

### Sensitivity analyses

A sensitivity analysis was performed by omitting blood cholesterol measurements as a predictor in the prediction models.

### Ethical approval

The University of Oxford Medical Sciences Inter-Divisional Research Ethics Committee approved the study. All individuals consented for the data collected at the screening to be used for research purposes.

### Data availability statement

Data from large population based studies conducted by the Nuffield Department of Population Health can be shared with bona fide researchers on application to the principal investigators of this study. Details of the departmental data access policy can be found at https://www.ndph.ox.ac.uk/data-access.

## Results

### Study population

The mean age in the derivation cohort was 62.2 ± 10.1 years and 35.7% were men. Overall, 12.2% of participants were current smokers and 28.2% were former smokers; 8.4% reported a history of DM. For prior vascular disease, 5.3% reported prior CHD, 3.4% stroke or TIA, and 2.3% PAD. The mean levels and proportions of cardiovascular risk factors and vascular disease were higher in participants with ACS than without ACS. The overall prevalence of ≥50% ACS was 1.9% and ≥70% ACS 0.3%. Baseline characteristics are shown in Table 1.

**Table 1:** Selected characteristics of participants screened for asymptomatic carotid stenosis (ACS) at baseline

	All participants (*n* = 596 469)	Participants with <50% ACS (*n* = 585 291)	Participants with 50%–69% ACS (*n* = 9 145)	Participants with ≥70% ACS (*n* = 2 033)∗
Age – y	62.2 ± 10.1	62.0 ± 10.0	68.7 ± 8.9	68.3 ± 8.8
Male sex	212 736 (35.7)	208 285 (35.6)	3 442 (37.6)	1 009 (49.6)
Current smoker	64 318 (12.2)	62 032 (12.0)	1 768 (22.2)	518 (28.8)
Ex-smoker	149 121 (28.2)	145 297 (28.0)	3 097 (38.8)	727 (40.4)
Never smoked	314 859 (59.6)	311 192 (60.0)	3 112 (39.0)	555 (30.8)
Diabetes mellitus	46 875 (8.4)	44 986 (8.2)	1 577 (18.3)	312 (16.4)
Stroke/TIA	18 186 (3.4)	17 154 (3.3)	758 (9.0)	274 (15.0)
CHD†	28 603 (5.3)	26 997 (5.1)	1 262 (14.9)	344 (18.6)
PAD	17 978 (3.1)	16 370 (2.8)	1 184 (13.4)	424 (21.8)
SBP – mmHg	132 ± 19.6	132 ± 19.5	142 ± 21.9	146 ± 23.5
DBP – mmHg	78 ± 9.8	78 ± 9.8	76 ± 10.2	78 ± 11.5
TC/HDL-C ratio	4.0 ± 1.6	4.0 ± 1.6	4.2 ± 1.7	4.4 ± 2.0
BMI – kg/m^2^	28.1 ± 5.4	28.1 ± 5.4	28.0 ± 5.3	27.9 ± 5.1
WC – cm	94.5 ± 15.5	94.4 ± 15.5	96.2 ± 14.9	97.9 ± 15.3
Aspirin	170 272 (33.5)	165 200 (33.1)	4 170 (52.4)	902 (52.8)
Lipid lowering therapy	151 831 (27.1)	146 845 (26.7)	4 065 (47.1)	921 (48.6)
Antihypertensives	197 396 (35.2)	191 112 (34.7)	5 115 (59.0)	1 169 (61.4)

Data are presented as *n* (%) or mean ± standard deviation. TIA = transient ischaemic attack; CHD = coronary heart disease; PAD = peripheral arterial disease; SBP = systolic blood pressure; DBP = diastolic blood pressure; TC = total cholesterol; HDL-C = high density lipoprotein cholesterol; BMI = body mass index; WC = waist circumference.

∗ In this group, 500 patients had a presumed occlusion.

† CHD is defined as previous myocardial infarction or a coronary intervention (bypass, angioplasty, or stenting).

### Risk score update and internal validation

Multivariable analyses demonstrated that all predictors used in the risk prediction model of de Weerd *et al.*^14^ were still significantly associated with ≥50% and ≥70% ACS, except for the association between DM and ≥70% ACS.^14^ PAD demonstrated a significant association with both outcomes and was included in the final risk score. SBP risk groups also demonstrated significant associations with both outcomes. Risks were higher in participants using antihypertensives than those not using antihypertensives in the same SBP risk groups (Table 2). In contrast, BMI and WC showed no improvement of risk prediction and were omitted from the final score.

**Table 2:** Multivariable predictors of moderate (*n* = 9 145) and severe (*n* = 2 033) asymptomatic carotid stenosis (ACS) in 596 469 screened individuals

	≥50% ACS	≥70% ACS	*p*
	OR (95% CI)	OR (95% CI)	
*Predictors*∗			
*Age (ref.: <50 y)*			
50–59	2.11 (1.80–2.48)	2.77 (1.84–4.18)	
60–69	4.09 (3.50–4.77)	5.18 (3.46–7.74)	
≥70	5.87 (5.02–6.86)	6.25 (4.16–9.39)	
Male sex	1.32 (1.27–1.37)	1.93 (1.76–2.11)	
Current smoking	2.69 (2.56–2.84)	3.07 (2.75–3.42)	
DM	1.37 (1.30–1.44)	1.07 (0.94–1.22)	
Stroke or TIA	1.69 (1.57–1.82)	2.47 (2.14–2.84)	
CHD	1.78 (1.68–1.89)	1.85 (1.63–2.10)	
PAD	2.85 (2.68–3.02)	3.90 (3.46–4.40)	
*SBP if not using antihypertensives (ref.: <125 mmHg)*			
<125	Ref.	Ref.	
125–139	1.69 (1.56–1.84)	1.90 (1.52–2.36)	
140–159	2.60 (2.39–2.83)	3.23 (2.60–4.01)	
≥160	4.41 (3.99–4.87)	6.30 (4.96–8.01)	
*SBP if using antihypertensives*			
<125	2.51 (2.31–2.74)	3.26 (2.62–4.05)	
125–139	2.91 (2.68–3.15)	3.19 (2.58–3.95)	
140–159	3.98 (3.67–4.32)	4.86 (3.95–5.96)	
≥160	5.81 (5.31–6.36)	8.46 (6.77–10.57)	
*DBP (ref.: ≥85 mmHg)*			
75–84	1.31 (1.23–1.39)	1.19 (1.04–1.35)	
<75	1.98 (1.86–2.12)	1.63 (1.41–1.88)	
TC/HDL-C ratio ≥ 5 (ref.: <5)	1.32 (1.26–1.38)	1.45 (1.31–1.60)	
Intercept†	−7.08	−9.40	
*Discrimination*			
AUROC after internal validation‡	0.78 (0.77–0.78)	0.82 (0.81–0.83)	
*Reclassification measures*			
IDI	0.011 (0.010–0.012)	0.006 (0.005–0.007)	<.001
rIDI	0.502 (0.463–0.542)	0.696 (0.555–0.842)	
NRI highest decile of predicted risk	0.346 (0.299–0.391)	0.491 (0.388–0.594)	<.001
NRI highest two deciles of predicted risk	0.305 (0.256–0.354)	0.376 (0.271–0.481)	<.001

Data are presented as odds ratio (OR) (95% confidence interval [CI]) unless stated otherwise. The original regression formula can be derived from the OR and the intercept. The beta coefficients of the linear predictor can be calculated by taking the natural logarithm of the ORs. The linear predictor function can be calculated with the following formula: $\text{LP}=\text{Intercept}+{\text{β}}_{1}{\text{x}}_{1}+{\text{β}}_{2}{\text{x}}_{2}+{\text{β}}_{3}{\text{x}}_{3}\ldots {\text{β}}_{\text{n}}{\text{x}}_{\text{n}},$where the β′s are the beta coefficients or weights of the predictors and the x's are the predictors. The predicted probability can be calculated by: $\frac{{\text{e}}^{\text{LP}}}{1+{\text{e}}^{\text{LP}}}$. Ref. = reference; DM = diabetes mellitus; TIA = transient ischaemic attack; CHD = coronary heart disease; PAD = peripheral arterial disease; SBP = systolic blood pressure; DBP = diastolic blood pressure; TC = total cholesterol; HDL-C = high density lipoprotein cholesterol; AUROC = area under receiver operating characteristic curve; IDI = integrated discrimination improvement; rIDI = relative integrated discrimination improvement; NRI = net reclassification improvement.

∗ Corrected for over optimism with bootstrapping techniques (shrinkage of regression coefficients was not necessary with calibration slope of 1.00).

† Bootstrap adjusted intercepts are reported. The intercept before internal validation was −7.08 for ≥50% ACS and −9.38 for ≥70% ACS.

‡ The AUROCs before internal validation were 0.78 (95% CI 0.77–0.78) for ≥ 50% ACS and 0.82 (95% CI 0.81–0.83) for ≥70% ACS.

**Figure 1:**
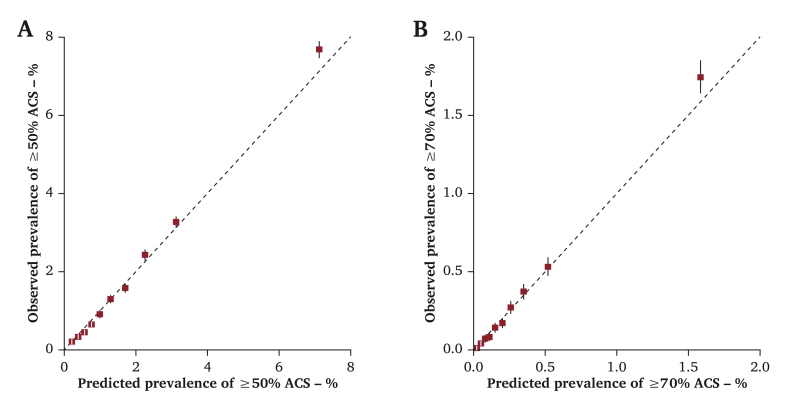
Calibration plot showing the mean predicted risk against the observed risk of (A) ≥ 50% and (B) ≥ 70% asymptomatic carotid stenosis (ACS) across deciles of predicted risk after internal validation. The boxes represent the mean predicted risk for each decile and the vertical lines represent the 95% confidence intervals. The dotted diagonal line indicates perfect calibration. Boxes above the diagonal line indicate underestimation of risk and below the diagonal line overestimation of risk. The prevalences and number of cases per decile are provided in Table S3 (see Supplementary Material).

The following predictors were included in the final risk score: age, sex, current smoking, DM, history of stroke/TIA, history of CHD, PAD, SBP (by use of antihypertensives), DBP, and TC/HDL ratio. The AUROC was 0.78 (95% confidence interval [CI] 0.77–0.78) for ≥ 50% ACS and 0.82 (95% CI 0.81–0.83) for ≥ 70% ACS. Internal validation with bootstrapping techniques indicated that no correction for over optimism of the beta coefficients was needed. Calibration plots showed a very good concordance between predicted and observed risk of both ≥50% and ≥70% ACS, indicating that groups of patients at both low and high risk can be predicted reliably by the risk score (Fig. 1).

### Clinical application and reclassification measures

**Figure 2:**
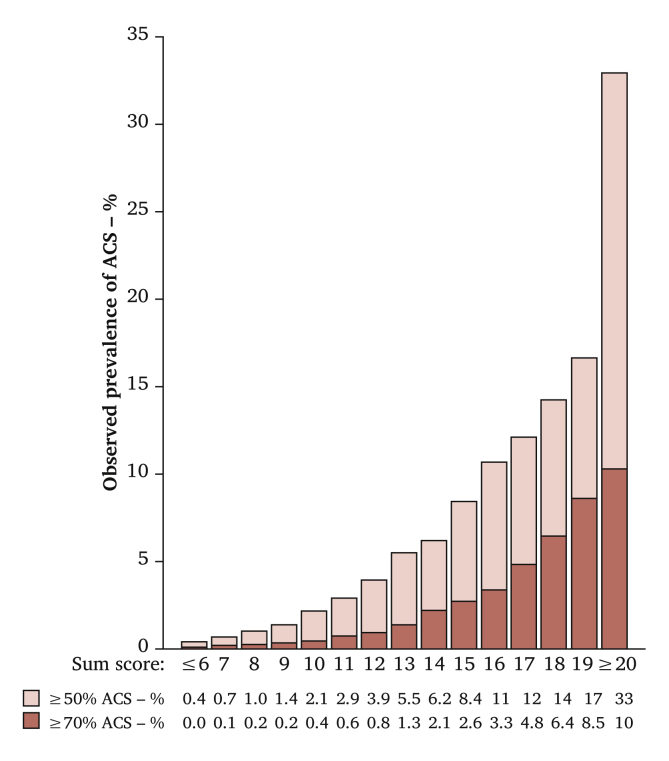
Bar chart showing the observed prevalence of asymptomatic carotid stenosis (ACS) for each sum score. The dark parts of the bars represent the prevalence of ≥70% ACS and the light parts the prevalence of 50%–69% ACS. The prevalence of ≥50% ACS is calculated by taking the sum of the prevalences of 50%–69% and ≥70% ACS.

**Figure 3:**
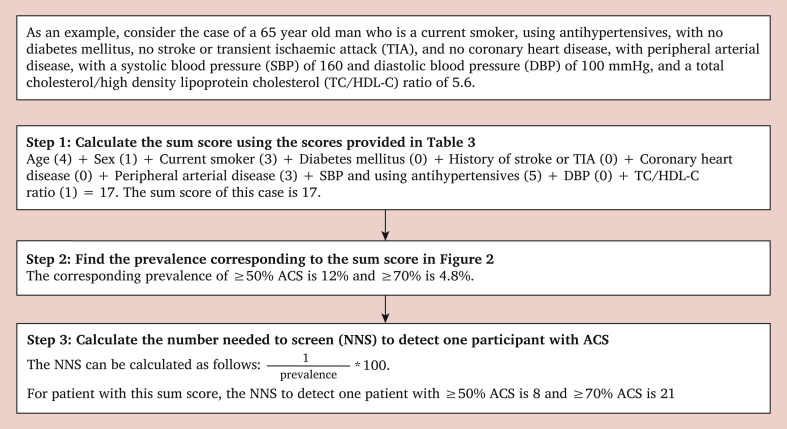
Calculating the risk of ≥50% and ≥70% asymptomatic carotid stenosis (ACS) using the Prevalence of Asymptomatic Carotid Artery Stenosis (PACAS) risk score. NNS = Number needed to screen.

The variables included in the risk score (PACAS score chart) are shown in Table 3. The scores ranged from 0 to 25. The risks of ≥50% and ≥70% ACS for each sum score are provided in Fig. 2. The calculation of the risk of ≥50% and ≥70% ACS for an example patient is provided in Fig. 3. The distribution of sum scores is provided in Figure S1 (see Supplementary Material).

**Table 3:** Predictors included in the Prevalence of Asymptomatic Carotid Artery Stenosis (PACAS) risk score and the associated scores

Predictors	Risk scores
*Age – y*	
<50	0
50–59	2
60–69	4
≥70	5
Male sex	1
Current smoking	3
Diabetes mellitus	1
History of stroke or TIA	2
Coronary heart disease	2
Peripheral arterial disease	3
*SBP if not using antihypertensives – mmHg*	
<125	0
125–139	2
140–159	3
≥160	4
*SBP if using antihypertensives – mmHg*	
<125	3
125–139	3
140–159	4
≥160	5
*DBP – mmHg*	
≥85	0
75–84	1
<75	2
*TC/HDL-C ratio*	
<5	0
≥5	1

The PACAS score ranges from 0 to 25. The risks of ≥50% and ≥70% asymptomatic carotid stenosis for each sum score are provided in Fig. 2. TIA = transient ischaemic attack; SBP = systolic blood pressure; DBP = diastolic blood pressure; TC = total cholesterol; HDL-C = high density lipoprotein cholesterol.

The prevalence of ≥50% ACS when screening the 10% highest risk participants was 7.7%. Hence, the NNS to detect one participant with ≥50% ACS was 13. A targeted screening of the 10% highest risk participants could identify 40.9% of cases with ≥50% ACS. The prevalence of ≥50% ACS when screening the 20% highest risk participants was 5.5%. Hence, the NNS to detect one participant with ≥50% ACS was 18. A targeted screening of the 20% highest risk participants could identify 58.4% of cases with ≥50% ACS (Table 4). The 10% highest risk corresponds approximately to targeted screening of participants with a sum score of ≥12 and the 20% highest risk corresponds approximately to targeted screening of participants with a sum score of ≥10.

**Table 4:** Performance of the Prevalence of Asymptomatic Carotid Artery Stenosis (PACAS) risk score to detect asymptomatic carotid stenosis (ACS) in 596 469 screened individuals

	Targeted screening of the 10% highest risk participants		Targeted screening of the 20% highest risk participants	
	≥50% ACS	≥70% ACS	≥50% ACS	≥70% ACS
No. of individuals screened	59 647	59 706	119 438	121 213
No. of cases with ACS	4 575	1 036	6 524	1 362
NNS	13	58	18	89
PPV/observed prevalence – %	7.7	1.7	5.5	1.1
NPV – %	98.8	99.8	99.0	99.9
Sensitivity – %	40.9	51.0	58.4	67.0
Specificity – %	90.6	90.1	80.7	79.8

Data are presented as *n* unless stated otherwise. The number of false negatives and true negatives were 6 603 and 530 219, and 4 654 and 472 377 for the highest decile and highest two deciles of predicted risk of ≥50% ACS, respectively, and, 997 and 535 766 and 671 and 474 585 for the highest decile and highest two deciles of predicted risk of ≥70% ACS, respectively. NNS = number needed to screen; PPV = positive predictive value; NPV = negative predictive value.

Reclassification measures demonstrated a significant improvement of the PACAS risk score compared with the risk score of de Weerd *et al.* (Table 2).^14^

### Sensitivity analysis

The discrimination of the internally validated PACAS risk score without inclusion of blood cholesterol measurements as predictors was 0.78 (95% CI 0.77–0.78) for ≥50% ACS and 0.82 (95% CI 0.81–0.82) for ≥70% ACS, respectively.

## Discussion

This risk score was developed to identify individuals at high risk of clinically significant ACS, which was defined as a stenosis that might alter clinical management. Predictors for moderate and severe ACS included age, sex, and vascular risk factors. Discrimination analysis was good for ≥50% ACS and even better for stenosis ≥70% ACS. Calibration plots showed reliable prediction of the prevalence of ≥50% and ≥70% ACS. The observed prevalence of ≥50% in the highest decile of predicted risk was 7.7%, with a NNS of 13; the observed prevalence of ≥70% in this decile was 1.7% with a NNS of 58. This new risk score outperformed existing risk scores by including additional predictors of ACS.

This risk score may contribute to a clinically and cost effective targeted screening protocol. Individuals in whom significant ACS is detected should receive intensive cardiovascular risk management, including lifestyle interventions and antihypertensive, antithrombotic, and lipid lowering drug therapy.^8^ Medical management not only aims to reduce the risk of stroke, but also reduce risks of other vascular disease, as ACS is also a risk factor for myocardial infarction and premature vascular death.^3^

Previous randomised trials that included a subset of individuals with ACS taking low dose aspirin reported an annual risk of ipsilateral stroke of between 1.4% and 2.4%.^19^, ^20^, ^21^ More recent studies reported an annual risk of ipsilateral ischaemic stroke of 0.34% in a cohort of individuals on intensive medical therapy after a TIA or minor stroke.^22^ Risks in asymptomatic individuals without a history of contralateral ischaemic cerebrovascular disease might be lower, but the intensity of medical prophylaxis is also often lower in such individuals. The present study showed considerable scope to further optimise medical therapy, with only around half of cases with ACS reporting the use of lipid lowering therapy and aspirin. While this was higher in patients in the highest decile of ≥50 ACS risk, with 76.4% reporting use of antihypertensives, 51.9% lipid lowering therapy, and 56.4% aspirin, only 30.2% used triple medical therapy. In the highest two deciles, 73.1% reported use of antihypertensives, 47.4% lipid lowering therapy, and 52.6% aspirin; only 25.7% used triple medical therapy.

A cost effectiveness analysis of this Swedish screening programme, where duplex ultrasound screening for abdominal aortic aneurysm was supplemented by ACS screening, suggested that the stroke risk could be reduced by 50% from antiplatelet and lipid lowering therapy combined, but with a wide margin of variation due to the absence of comparative studies.^23^^,^^24^

Predictors of increased stroke risk in individuals with ACS have been identified and carotid interventions might be considered in patients in whom the absolute gain of reducing the risk of stroke by carotid revascularisation are found to be worthwhile.^8^ Use of imaging characteristics and risk stratification tools for individualised prediction of stroke based on multiple predictors have been proposed, but these have not been validated in independent contemporary populations.^8^^,^^25^^,^^26^

The PACAS risk score can both be applied to cases with and without overt cardiovascular disease and could be used for targeted screening. Such risk prediction can be performed easily by general practitioners and specialists with the aim of initiating or intensifying cardiovascular risk management. Different imaging modalities and criteria for measuring stenosis of the carotid arteries are available. The validity of duplex ultrasound is good if performed by experienced sonographers.^27^ PSV as a single measure may be useful as a screening tool to identify cases for more detailed evaluation.

The derivation cohort used in the present study has several limitations, including the fact that participants were self referred and self funded, which might influence the generalisability to other populations. Although a large number of participants with lower scores was found in the current population, the magnitude of multivariable predictors of ACS was similar to previous population based studies.^14^ Duplex ultrasounds were not performed in validated ultrasound laboratories by qualified sonographers, but simplified screening methods showed reasonable interobserver reliability and validity.^28^^,^^29^ Recall bias cannot be fully excluded for predictors that were self reported. Blood pressure and cholesterol were measured once and might not reflect “usual” values. Indications for duplex ultrasound were not available and recommended treatment for patients in whom carotid artery occlusion is found might differ from patients with carotid stenosis. Clinical staging of PAD was not available. These limitations indicate the need for validation of the risk score in an external population. Even though the NNS was greatly reduced in high risk cases *vs.* population level screening, the positive predictive value indicates that many cases considered high risk will have no ACS. Past medication use and potential reasons for quitting were not recorded.

Despite these limitations, the present study has some important strengths, including the use of a relatively contemporary derivation cohort of 0.6 million participants for the development of the PACAS risk score, and internal validation showed no evidence for overfitting. The sensitivity analysis showed that risk prediction based on patient characteristics and measured blood pressure is equally reliable and that excluding blood cholesterol measurement does not affect adequate risk predictions. Application of the PACAS risk score and calculation of individualised risks can be done quite easily.

Future research will establish the optimal threshold for targeted screening by determining the risks of stroke and other cardiovascular diseases in ACS cases and how many such cardiovascular events could be prevented by improved cardiovascular risk management in patients in whom ACS is detected using a cost effective targeted screening programme. Whether screening for ACS can be combined with screening for other risk factors for stroke, such as atrial fibrillation, possibly further reducing stroke incidence, should be determined.

### Conclusions

The novel PACAS risk score, including age, sex, current smoking, DM, history of stroke/TIA, CHD and PAD, SBP (by use of antihypertensives), DBP, and TC/HDL ratio, can predict the risk of ≥50% and ≥70% ACS reliably, and performed better than other risk scores. The prevalences in the decile at highest predicted risk of ≥50% and ≥70% ACS were 7.7% and 1.7%, respectively. Targeted screening of this high risk group identified over one third (41%) of cases with ≥50% ACS, with a NNS of 13, and over half (51%) of cases with ≥70% ACS, with a NNS of 58.

## Conflicts of interest

This study was designed, conducted, and reported independently of Life Line Screening, who provided their data and all sources of support at no cost.

## Funding

Alison Halliday is funded by the 10.13039/501100000272UK Health Research (NIHR) 10.13039/501100013373Oxford Biomedical Research Centre (BRC). Sarah Lewington is funded by the 10.13039/501100000265UK Medical Research Council and the CDC foundation (with support from Amgen).
