# Supplementary material for: Development and Internal Validation of a Risk Score to Detect Asymptomatic Carotid Stenosis
Source: Eur J Vasc Endovasc Surg. 2021 Mar;61(3):365–73. doi: 10.1016/j.ejvs.2020.11.029 (PMC7994015; doi:10.1016/j.ejvs.2020.11.029)
Supplement: Multimedia component 1 [file mmc1.pdf]

Development and internal validation of a risk score to detect asymptomatic carotid stenosis – Supplementary material

Supplemental Table 1. Missing data per variable

Variable	Percentage of participants with missing
Age	0
Sex	0
Smoking status	11.4
Diabetes mellitus	6.2
Stroke/TIA	9.9
Coronary heart disease	8.9
Peripheral arterial disease	1.3
SBP	0.5
DBP	31.8
TC/HDL-ratio	0.3
BMI	4.1
WC	34.9
Antihypertensives	5.9

BMI, body mass index; DBP indicates diastolic blood pressure; HDL-C, high-density lipoprotein cholesterol; LDL-C, low-density lipoprotein cholesterol; SBP, systolic blood pressure; TC, total cholesterol; TIA, transient ischemic attack; WC, waist circumference.

Supplemental Table 2 TRIPOD Checklist

Section/Topic	Item	Checklist Item	Page
**Title and abstract**			
Title	1	Identify the study as developing and/or validating a multivariable prediction model, the target population, and the outcome to be predicted.	✓ Title
Abstract	2	Provide a summary of objectives, study design, setting, participants, sample size, predictors, outcome, statistical analysis, results, and conclusions.	✓ Abstract
**Introduction**			
Background and objectives	3a	Explain the medical context (including whether diagnostic or prognostic) and rationale for developing or validating the multivariable prediction model, including references to existing models.	✓ Intro
	3b	Specify the objectives, including whether the study describes the development or validation of the model or both.	✓ Intro
**Methods**			
Source of data	4a	Describe the study design or source of data (e.g., randomized trial, cohort, or registry data), separately for the development and validation data sets, if applicable.	✓ M&M
	4b	Specify the key study dates, including start of accrual; end of accrual; and, if applicable, end of follow-up.	✓ M&M
Participants	5a	Specify key elements of the study setting (e.g., primary care, secondary care, general population) including number and location of centres.	✓ M&M
	5b	Describe eligibility criteria for participants.	✓ M&M
	5c	Give details of treatments received, if relevant.	NA
Outcome	6a	Clearly define the outcome that is predicted by the prediction model, including how and when assessed.	✓ M&M
	6b	Report any actions to blind assessment of the outcome to be predicted.	NA
Predictors	7a	Clearly define all predictors used in developing or validating the multivariable prediction model, including how and when they were measured.	✓ M&M
	7b	Report any actions to blind assessment of predictors for the outcome and other predictors.	NA
Sample size	8	Explain how the study size was arrived at.	✓ M&M
Missing data	9	Describe how missing data were handled (e.g., complete-case analysis, single imputation, multiple imputation) with details of any imputation method.	✓ M&M
Statistical analysis methods	10a	Describe how predictors were handled in the analyses.	✓ M&M
	10b	Specify type of model, all model-building procedures (including any predictor selection), and method for internal validation.	✓ M&M
	10d	Specify all measures used to assess model performance and, if relevant, to compare multiple models.	✓ M&M
Risk groups	11	Provide details on how risk groups were created, if done.	✓ M&M
**Results**			
Participants	13a	Describe the flow of participants through the study, including the number of participants with and without the outcome and, if applicable, a summary of the follow-up time. A diagram may be helpful.	✓ M&M
	13b	Describe the characteristics of the participants (basic demographics, clinical features, available predictors), including the number of participants with missing data for predictors and outcome.	✓ Results
Model development	14a	Specify the number of participants and outcome events in each analysis.	✓ Results
	14b	If done, report the unadjusted association between each candidate predictor and outcome.	NA
Model specification	15a	Present the full prediction model to allow predictions for individuals (i.e., all regression coefficients, and model intercept or baseline survival at a given time point).	✓ Table 2
	15b	Explain how to the use the prediction model.	✓ Results
Model performance	16	Report performance measures (with CIs) for the prediction model.	✓ Results
**Discussion**			
Limitations	18	Discuss any limitations of the study (such as nonrepresentative sample, few events per predictor, missing data).	✓ Discussion

Interpretation	19b	Give an overall interpretation of the results, considering objectives, limitations, and results from similar studies, and other relevant evidence.	✓ Discussion
Implications	20	Discuss the potential clinical use of the model and implications for future research.	✓ Discussion
**Other information**			
Supplementary information	21	Provide information about the availability of supplementary resources, such as study protocol, Web calculator, and data sets.	✓ Appendix
Funding	22	Give the source of funding and the role of the funders for the present study.	✓

Supplemental Table 3. Predicted and observed prevalence of ACS across deciles of predicted risk

	Deciles of predicted risk of ≥50% ACS									
**Predicted prevalence based on PACAS score (%)**	0.21	0.38	0.56	0.75	0.99	1.29	1.70	2.25	3.12	7.14
**Observed prevalence (%)**	0.20	0.32	0.44	0.64	0.90	1.29	1.57	2.42	3.26	7.67
**Number of cases with ≥50% ACS**	114	198	267	378	526	796	938	1437	1949	4575

ACS, asymptomatic carotid artery stenosis; PACAS score, Prevalence of Asymptomatic Carotid Artery Stenosis score.

	Deciles of predicted risk of ≥70% ACS									
**Predicted prevalence based on PACAS score (%)**	0.02	0.05	0.08	0.11	0.15	0.20	0.26	0.35	0.52	1.59
**Observed prevalence (%)**	0.01	0.04	0.07	0.08	0.14	0.17	0.27	0.37	0.53	1.74
**Number of cases with ≥70% ACS**	4	24	41	48	81	101	160	212	326	1036

ACS, asymptomatic carotid artery stenosis; PACAS score, Prevalence of Asymptomatic Carotid Artery Stenosis score.

Supplemental Figure 1.

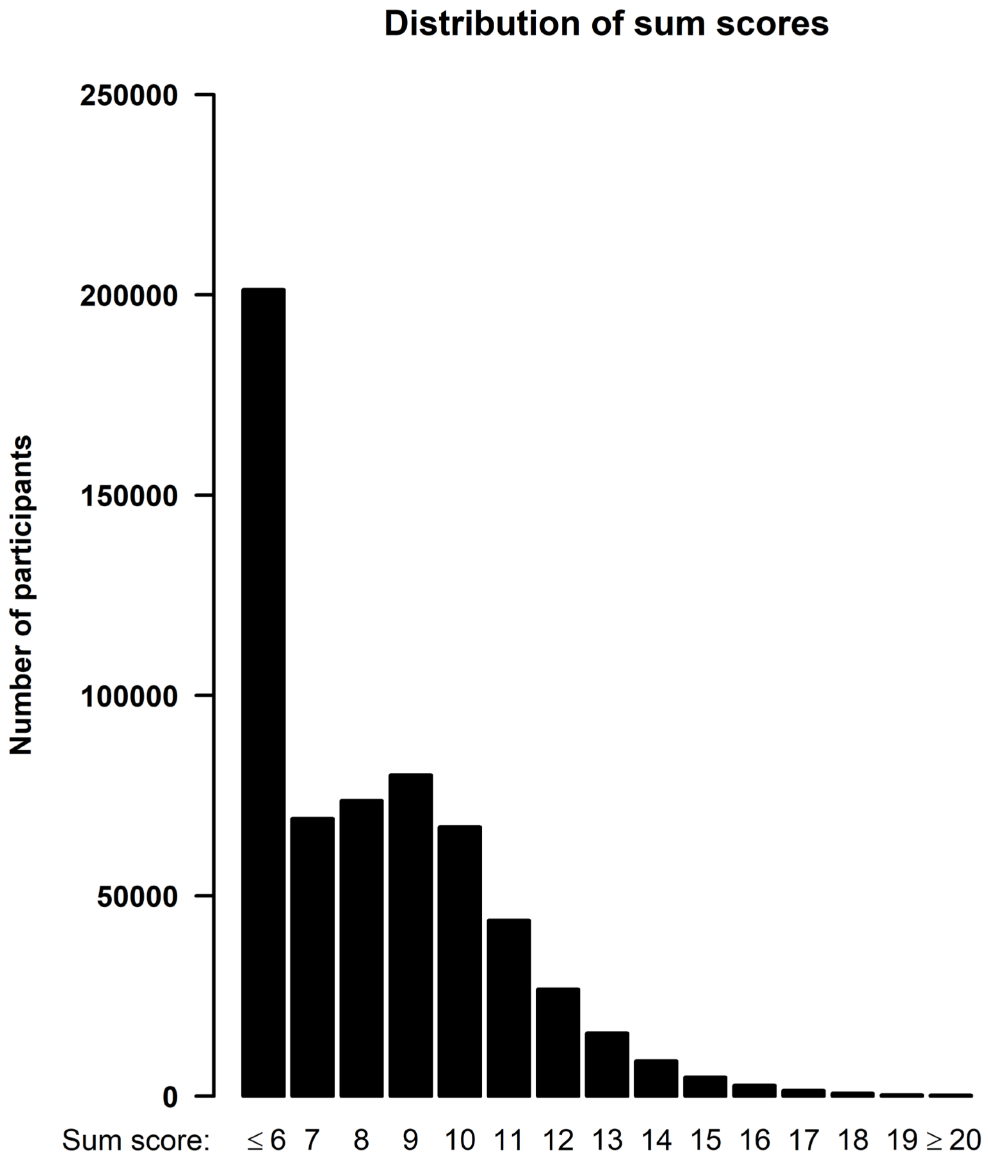

Supplemental Methods 1.

Additional description of the imputation

For imputation of missing values, we used chained equations and we created 20 datasets with 200 iterations.[1] TC/HDL-ratio was calculated before imputation.[2] Post-imputation rounding was applied for limited-range variables (body mass index (BMI), WC, SBP, DBP, and TC/HDL-ratio), if needed.[3] Analyses were performed in the resulting 20 imputed datasets and results were pooled using Rubin's rules.[4, 5]

Additional description of model development

We performed re-estimation of the intercept and predictor weights (beta-coefficients) of the predictors included in the risk score developed by de Weerd et al.[6] These predictors included age groups, sex, current smoking, diabetes mellitus, history of stroke or myocardial infarction, SBP groups, DBP groups, TC/HDL ratio groups. We also performed model extension with forward stepwise selection with predictors selected using Akaike Information Criterion (AIC).[7] For this, we tested whether adding PAD, BMI, and WC improved prediction, as well whether an additional risk group for SBP and SBP by use of antihypertensives improved prediction.

Additional description of internal validation

Bootstrap techniques were used for internal validation. We created 1000 bootstrap replications per imputed dataset.[8] We calculated the mean calibration slope, a measure that reflects the extent of overfitting, of the 1000 bootstrap replications in each imputed dataset used that as a uniform shrinkage factor to adjust the regression coefficients for risk of potential overfitting.[9] We used the shrunken beta-coefficients to calculate the adjusted intercept by fitting a logistic model with the shrunken beta-coefficients as dependent variables in the original dataset. We calculated overoptimism-corrected AUROC for each imputed dataset and combined the results with Rubin's rules.[4, 5]

Additional description of reclassification measures

We calculated predicted probabilites of ≥50% and ≥70% using the original risk equation provided by de Weerd et al, 2014 (called the 'original' model) and calculated reclassification measures to assess the

ability of our novel model (called the 'updated' model) to correctly identify cases in comparison with the original model.[6]

We calculated integrated discrimination improvement (IDI), relative IDI (rIDI), and category-based net reclassification improvement (NRI).[10, 11] IDI is the absolute difference in discrimination slopes of the updated and original model. rIDI is the ratio of absolute difference in discrimination slopes of the updated and original model over the discrimination slope of the original model. Category-based NRI is the proportion of individuals correctly reclassified with the updated risk score across risk categories (in this study, the highest decile and highest two deciles of predicted risk) minus the proportion of individuals incorrectly reclassified. Positive values correspond to improved classification.

The reclassification measures were estimated for all 1000 bootstrap replications in each imputed dataset and the median value across the combined 20 datasets is reported (with the 95% confidence interval obtained from the 2.5th and 97.5th percentiles). *P* values <0.05 were considered significant.

Reference list

1. White IR, Royston P, Wood AM. Multiple imputation using chained equations: Issues and guidance for practice. *Stat Med*. 2011;30:377-399
2. Morris TP, White IR, Royston P, Seaman SR, Wood AM. Multiple imputation for an incomplete covariate that is a ratio. *Stat Med*. 2014;33:88-104
3. Rodwell L, Lee KJ, Romaniuk H, Carlin JB. Comparison of methods for imputing limited-range variables: A simulation study. *BMC Med Res Methodol*. 2014;14:57
4. Marshall A, Altman DG, Holder RL, Royston P. Combining estimates of interest in prognostic modelling studies after multiple imputation: Current practice and guidelines. *BMC Med Res Methodol*. 2009;9:57
5. Rubin DB. Inference and missing data. *Biometrika*. 1976;63:581-592
6. de Weerd M, Greving JP, Hedblad B, Lorenz MW, Mathiesen EB, O'Leary DH, et al. Prediction of asymptomatic carotid artery stenosis in the general population: Identification of high-risk groups. *Stroke*. 2014;45:2366-2371
7. Akaike H. Statistical predictor identification. *Ann Inst Stat Math*. 1970;22:203-217
8. Steyerberg EW, Harrell FE, Jr., Borsboom GJ, Eijkemans MJ, Vergouwe Y, Habbema JD. Internal validation of predictive models: Efficiency of some procedures for logistic regression analysis. *J Clin Epidemiol*. 2001;54:774-781
9. Steyerberg EW. *Clinical prediction models: A practical approach to development, validation, and updating*. New York: Springer-Verlag 2009.
10. Pencina MJ, D'Agostino Sr. RB, D'Agostino Jr. RB, Vasan RS. Evaluating the added predictive ability of a new marker: From area under the roc curve to reclassification and beyond. *Stat Med*. 2008;27:157-172
11. Leening MJ, Vedder MM, Witteman JC, Pencina MJ, Steyerberg EW. Net reclassification improvement: Computation, interpretation, and controversies: A literature review and clinician's guide. *Ann Intern Med*. 2014;160:122-131
